# Thermal activation energy on electrical degradation process in BaTiO_3_ based multilayer ceramic capacitors for lifetime reliability

**DOI:** 10.1038/s41598-024-51254-w

**Published:** 2024-01-05

**Authors:** Jinsung Chun, Jungwoo Heo, KyungSoo Lee, Byeong Uk Ye, Byung Sung Kang, Seok-Hyun Yoon

**Affiliations:** 1 MLCC Development Team, Component Biz. Unit, Samsung Electro-Mechanics Co. Ltd., Gyunggi-Do, Suwon, 16674 Republic of Korea; 2 Development QA Team, Quality Assurance Center, Samsung Electro-Mechanics Co. Ltd., Gyunggi-Do, Suwon, 16674 Republic of Korea; 3 MLCC Manufacturing Technology Team, Component Biz. Unit, Samsung Electro-Mechanics Co. Ltd., KangSeo-Ku, Pusan, 46754 Republic of Korea

**Keywords:** Electronic devices, Electrical and electronic engineering

## Abstract

For a high capacitance and high lifetime reliability of multilayer ceramic capacitors for automotive applications, the activation energy on thermal activation process can typically be calculated by using Arrhenius based Prokopowicz–Vaskas equation as a method for lifetime prediction. In this study, it is clearly observed that the activation energy shows to be constant in the range of ~ 1.5 eV for the prototype MLCCs, higher than the activation energy values of ~ 1.0 eV related to the motion or diffusion of oxygen vacancies reported in the previous literature. The activation energy value of ~ 1.5 eV for three prototype MLCCs is close to a half the energy band gap (*E*_g_/2 ≈ 1.6 eV) of BaTiO_3_ obtained from specific environment, where oxygen vacancies are stabilized by external containment such as the effect of rare earth oxide additives. Due to an obvious difference in activation energy values, it difficult to explain the conduction mechanism for failure by only oxygen vacancy migration. Therefore, the concepts of electronic processes and oxygen vacancy should be considered together to understand conduction mechanism for failure of BaTiO_3_-based MLCCs in thermal activation processes. It can be useful as an indicator for future MLCC development with high lifetime reliability.

## Introduction

Multilayer ceramic capacitors (MLCCs) have been an important passive component in the expanding market for electronic products such as laptops, tablets, and smartphones. Recently, the demand for components with high lifetime reliability used in harsh environments has increased dramatically due to the development of autonomous driving and electric vehicles^1^. In the case of MLCCs used in automobile applications, warranty conditions have significantly higher temperature and voltage levels than those used in general electronics. The life expectancy calculated under accelerated conditions calls for a lifespan that is approximately 50 times higher than the average lifespan of an IT application^2^.

To meet these demands and improve the lifetime of MLCC, a typical method have been approached from dispersing and mixing barium titanate (BaTiO_3_) with various additives of rare earth oxides. (i.e., Dy, Y, Ho, Yb, Er, etc.) Among them, Dy has long been known to play the most important role as a donor to improve insulation resistance and lifetime reliability^3–6^. Furthermore, when the rare earth elements are added to the BaTiO_3_ ceramic used as the base material for MLCCs, the role of rare earth elements is well known to inhibit the formation and the migration of oxygen vacancies leading to a major cause of failure^2,7^.

1 $$\text{Ba site} : {{\text{R}}}_{2}{{\text{O}}}_{3} \to 2{{\text{R}}}_{{\text{Ba}}}^{*}+ {{\text{V}}}_{{\text{Ba}}}^{"}+3{{\text{O}}}_{{\text{O}}}$$

Equation (1) is a defect equation using the Kroger-Vink diagram to substitute the rare earth element Dy for the element Ba at the A-site. It can be explained that Dy can act as a donor and significantly improve lifetime reliability by suppressing oxygen vacancies leading to a major cause of defects due to + 1 valence of Dy than Ba. This approach has been shown to cause MLCCs to fail in experiments by a number of researchers since the 1990s^4^.

However, recent studies have a different opinion in comparison to the conventional hypothesis that oxygen vacancies are a major factor for the conduction mechanism in BaTiO_3_ in spite of suppression of oxygen vacancies by the effect of rare earth oxide additives. Even though higher activation energies have been reported in recent studies of MLCCs, it has been referred that band shifts or electron polaron hopping associated with oxygen vacancies are a major factor. The previous concept of mobile oxygen vacancies was issued for understanding the electrical behavior of perovskite oxide ferroelectrics, because it mimics the phenomenon of oxygen vacancy migration^8,9^. Therefore, we propose that the failure mechanisms explained solely by the oxygen vacancy migration in BaTiO_3_ based MLCCs should be reconsidered from an electronic perspective.

Here, we evaluate the lifetime of the prototype MLCCs using high-acceleration life testing (HALT) to obtain the mean time to failure (MTTF). The MTTF is used as a key factor to calculate the thermal activation energy of the MLCC with Arrhenius based Prokopowicz–Vaskas (P–V) equation by comparing it with values reported in previous papers. The activation energy values in the thermal activation process of the prototype MLCCs were investigated and compared to previous values based on the mobile oxygen vacancy concept. From these results, we discuss the failure mechanism of MLCCs with temperature acceleration in relation to the electronic process rather than the similar motion of oxygen vacancies.

## Experimental

**Figure 1:**
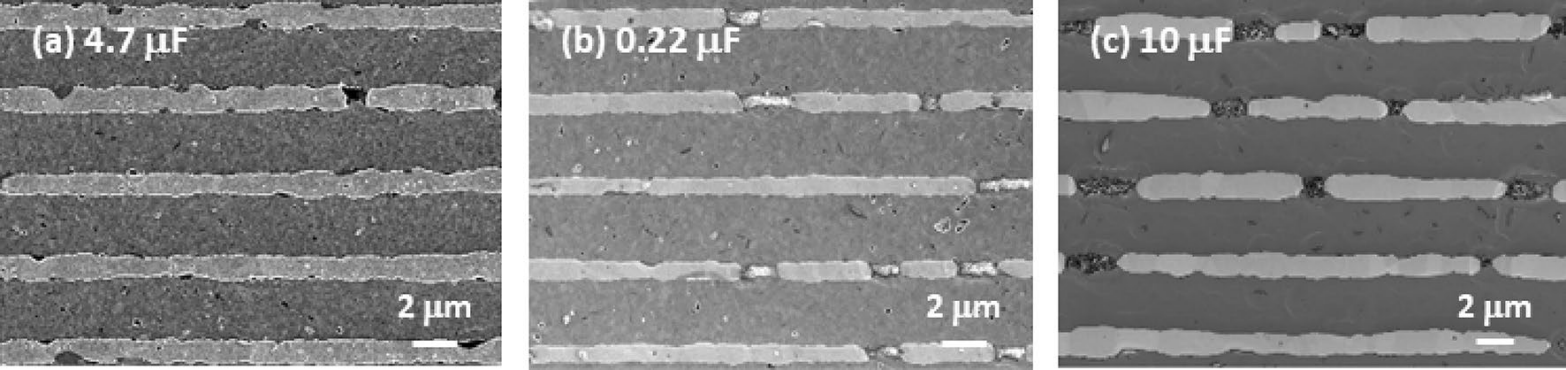
Cross-sectional SEM images of the (**a**) 4.7 μF, (**b**) 0.22 μF, and (**c**) 10 μF MLCCs used in this study.

Three prototypes of rare-earth-doped BaTiO_3_-based MLCCs (4.7 μF, 0.22 μF, and 10 μF) with the highest rated voltage (global standard) for automotive applications were used in this paper. Table 1 summarizes the specifications of capacitance, temperature characteristics, rated voltage, and dielectric thickness. The microstructures of the three MLCCs were examined using a scanning electron microscope to obtain average dielectric layer thickness. The cross-sectional SEM images of the 4.7 μF, 0.22 μF, and 10 μF MLCCs used in this study are shown in Fig. 1.

**Table 1:** Specifications of the prototype MLCCs for automotive applications used in this work.

Sample	Size	Capacitance (μF)	Temperature range (°C)	Capacitance change (%)	Rated voltage (V)	Dielectric thickness (μm)
4.7 μF MLCC	2012	4.7	– 55–125	± 22	50	4.2
0.22 μF MLCC	1005	0.22	– 55–125	± 15	25	4.5
10 μF MLCC	2012	10	– 55–125	± 22	25	3.2

In order to calculate MTTF of the MLCCs, all electrical measurements were conducted in a custom-made apparatus with a power supply system, a chamber with temperature controller, and a digital multi meter (DMM 7510, Keithley). The time-dependent insulation resistance (IR) degradation of MLCCs was evaluated under a constant dielectric current (DC) bias (70 V, 80 V and 95 V) for HALT as function of temperature ranges from 140 to 160 °C up to ~ 1500 h. To show successful introduction of Dy (rare-earth metal) into grains of dielectric layer, the high-resolution transmission electron microscopy (HR-TEM) is used (Tecnai Osiris 200 kV, FEI, USA).

## Result and discussion

**Figure 2:**
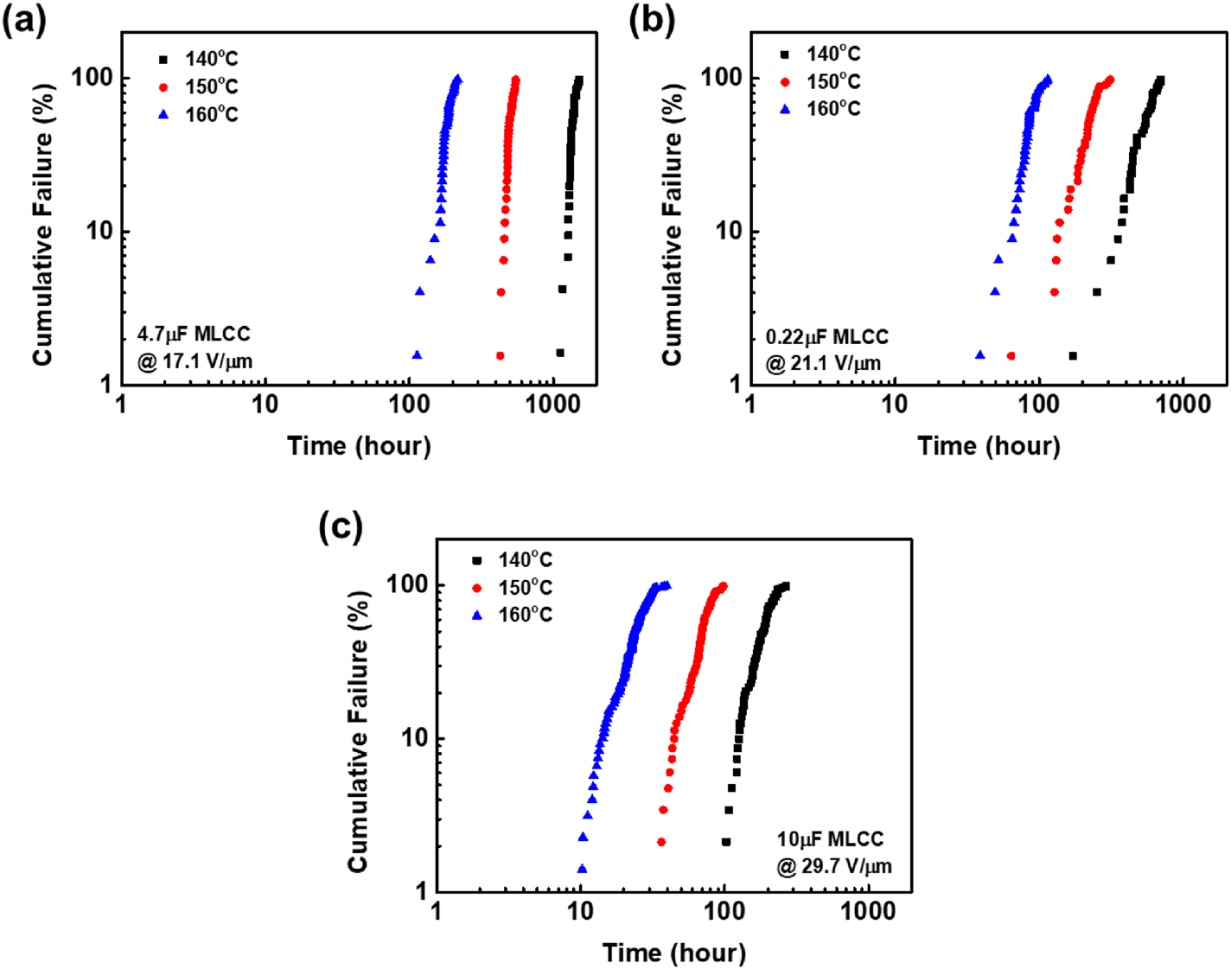
Weibull distribution for HALT results as a function of temperature from 140 to 160 °C at the constant electric field of 17.1 V/μm for (**a**) 4.7μF MLCC, 21.1 V/μm for (**b**) 0.22μF MLCC and 29.7 V/μm for (**c**) 10μF MLCC for ~ 1500 h.

Weibull plots for the time to failure at each temperature are shown in Fig. 2, showing MTTFs of the three prototype MLCCs (4.7 μF, 0.22 μF, and 10 μF) after HALT evaluation. Overall, it is clearly seen that time to failure decreases with increase of temperature from 140 to 160 °C and electric field from 17.1 to 29.7 V/μm, regardless of capacitance. It is found that the temperature and electric field applied to the dielectric are critical for lifetime of MLCCs. To calculate MTTF of the MLCCs from the time to failure, the two-parameter statistical Weibull distribution model was used. MTTF can be obtained from the following equation:

2 $$MTTF = \eta \Gamma \left( {1 + \beta^{ - 1} } \right)$$

where *β* is the dimensionless slope parameter for characteristic of the particular failure mode, *η* is the scale parameter, which represents the characteristic time at the population of the failed specimens reaching 63.2%, and *Γ* is the gamma function (Note: *Γ*(1 + *β*^-1^)  ≈ 0.9, when *β* > 3.0)^10^.

**Figure 3:**
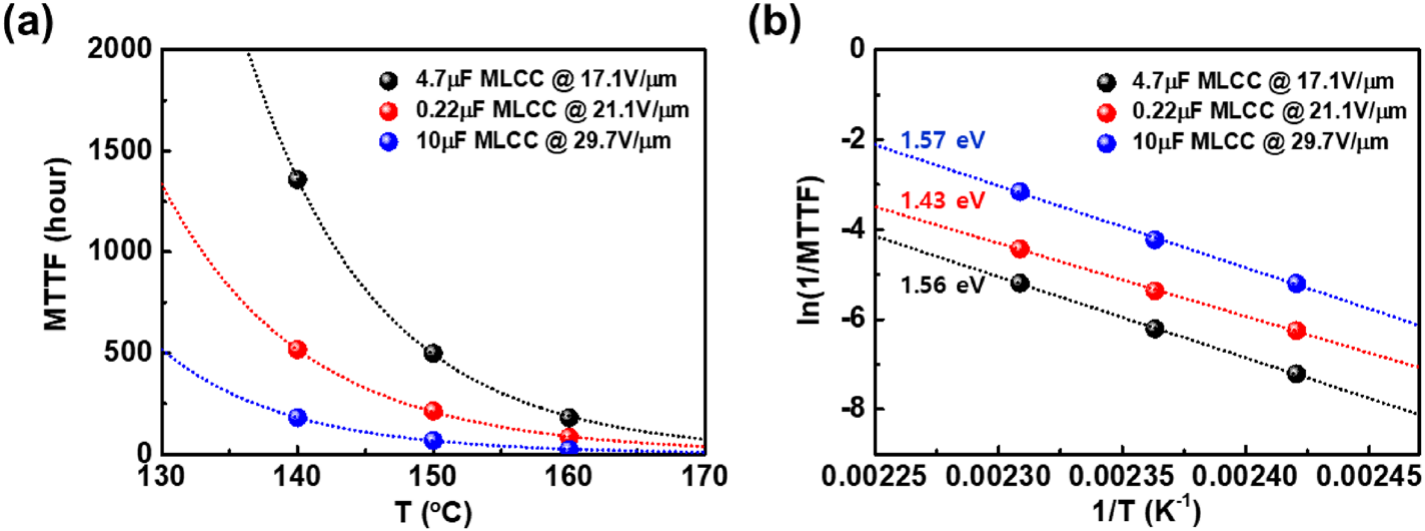
(**a**) The calculated MTTFs of the MLCCs (4.7μF, 0.22μF, and 10μF) and (**b**) Arrhenius plot for the MTTFs under temperature-accelerated lifetime test as a function of temperature from 140 to 160 °C at the constant electric field of 17.1 V/μm for 4.7μF MLCC, 21.1 V/μm for 0.22μF MLCC and 29.7 V/μm for 10μF MLCC for ~ 1500 h. Fit lines can be calculated by Arrhenius equation to obtain the activation energy.

The calculated MTTFs of the three MLCCs (4.7 μF, 0.22 μF, and 10 μF) are shown in Fig. 3a. It is obvious that the MTTF of the MLCCs increases exponentially with decreasing temperature from 160 to 140 °C. It is clearly shown that the higher the test temperature, the smaller the MTTF of MLCC. The empirical equation suggested by Prokopowicz and Vaskas (P–V) is commonly employed in order to predict the use-level lifetime of MLCCs as follows^11,12^:

3 $$A = \frac{{t_{1} }}{{t_{2} }} = \left( {\frac{{V_{2} }}{{V_{1} }}} \right)^{n} exp\left[ {\frac{{E_{a} }}{{k_{B} }}\left( {\frac{1}{{T_{1} }} - \frac{1}{{T_{2} }}} \right)} \right],$$

where *A* is the ratio of two times-to-failure or the acceleration factor in relation to time-to-failure *t*_1_ (h) under the first condition in voltage *V*_1_ (V) and temperature *T*_1_ (K) to time-to-failure *t*_2_ under the second condition in *V*_2_ and *T*_2_. *E*_a_ is the activation energy (eV), *n* is voltage acceleration constant, and *k*_B_ is the Boltzmann constant (eV/K) for HALT. When the voltage is constant, Eq. (3) can be expressed as the following:

4 $$\frac{1}{MTTF} = K_{0} exp\left[ { - \frac{{E_{a} }}{{k_{B} T}}} \right],$$

where *K*_0_ is the degradation rate constant. Equation (4) means thermal activation process related to dielectric breakdown for HALT of MLCC^2,13,14^. Figure 3b shows Arrhenius plot for the MTTF data with a fit line using Arrhenius-type Eq. (4). As depicted in Fig. 3b, the activation energy estimated from the Arrhenius-type equation fitting can exhibit an average value of 1.52 eV for all MLCCs, higher than the activation energy values of ~ 0.9 eV first reported by paper to use the P–V equation^15^. The 1.52 eV value in this study is especially distinguished from the activation energy value of 1.0 eV proposed by Waser et al. due to a phenomenon caused by oxygen vacancy migration^7^. The agreement of these experimental values led to the interpretation that the main cause of failure in MLCCs is related to oxygen vacancy migration. However, recent products developed for high lifetime reliability exhibit a relatively high activation energy, which converges to ~ 1.6 eV as in this paper. Due to an obvious difference in activation energy values, it difficult to explain the conduction for failure by only oxygen vacancy migration.

**Figure 4:**
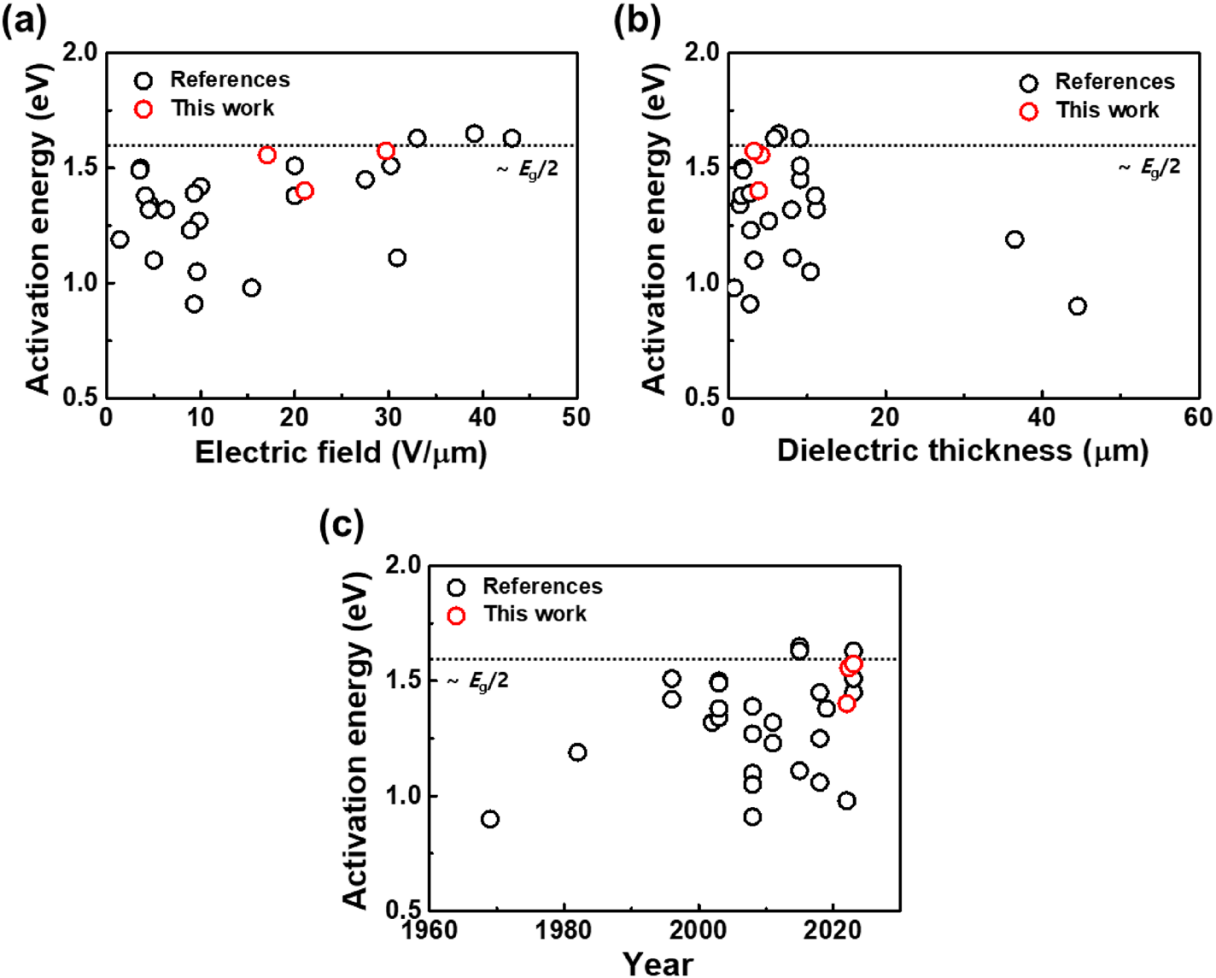
Activation energy in references^2,11–13,15–21^ and this work as functions of (**a**) electric field from 1.4 to 29.7 V/μm during HALT, (**b**) dielectric thickness from 1.4 to 44.5 μm of MLCC and (**c**) published year from 1696 to present.

Figure 4 shows the activation energy calculated from the reference^2,11–13,15–21^ and this work as functions of electric field during HALT, dielectric thickness of MLCC, and published year since 1969. As the electric field during HALT increases, the activation energies in references and this work are close to a constant value of ~ 1.6 eV, which is close to a half the energy band gap^22^ (*E*_g_/2 ≈ 1.6 eV) of BaTiO_3_ as shown in Fig. 4a. In case of the prototype MLCCs, the high electric field means that higher manufacturing skills for materials, equipment, etc. are required in comparison to conventional MLCCs of the past. It is also seen that the thinner the dielectric thickness, the more values close to 1.6 eV as shown in Fig. 4b. Since 1969, activation energy in chronological order is shown in Fig. 4c. The detailed reference data corresponding to Fig. 4 is summarized in Table S1 in Supplementary information.

**Figure 5:**
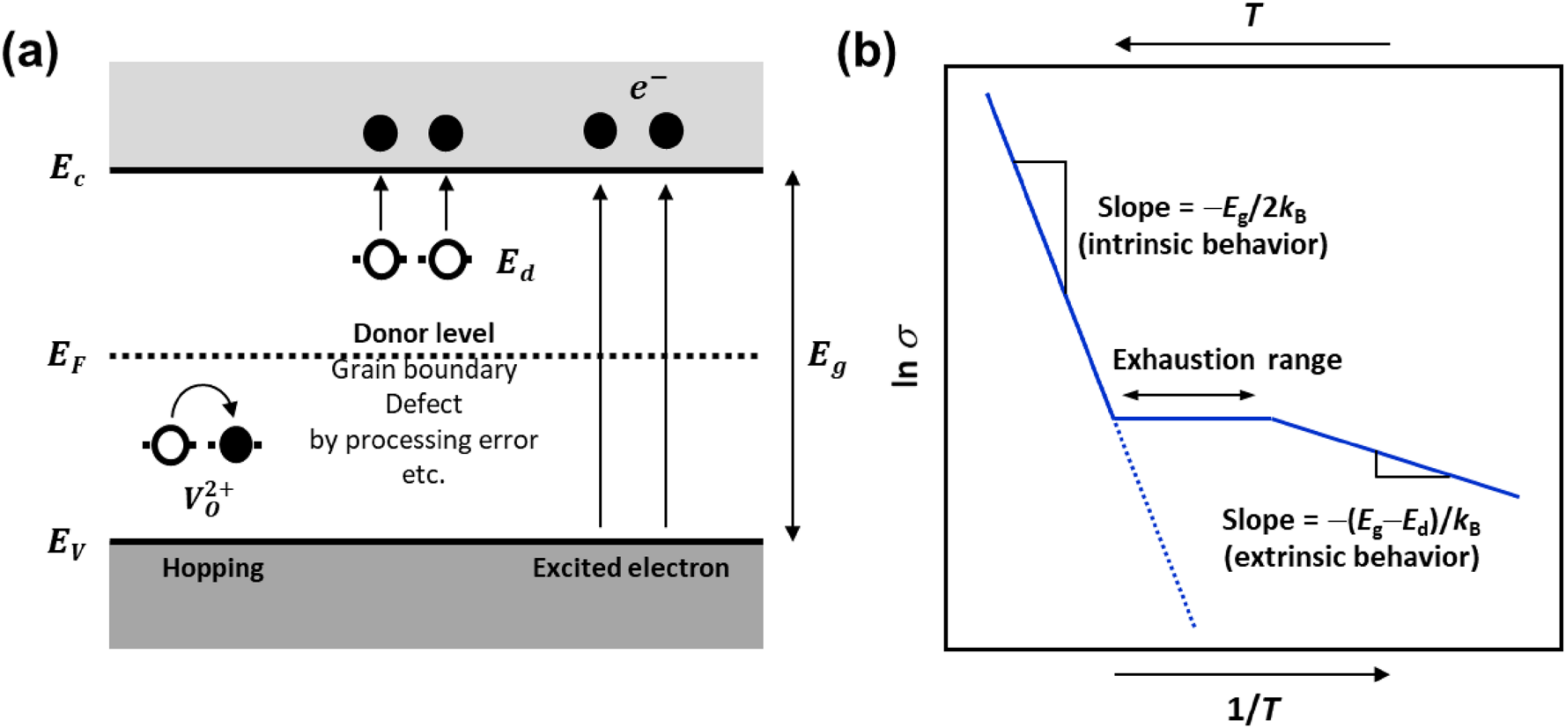
(**a**) Schematic images of simplified band diagrams for pure BaTiO_3_ showing oxygen vacancy related polaron hopping process and excited electron based on donor level/band gap in electronic process under thermal activation or electric field. (**b**) Arrhenius plot of electrical conductivity for an *n*-type semiconductor as a function of temperature^26^.

From the difference in activation energy, we propose to explain the activation energy by the conduction mechanism of BaTiO_3_, based on the concept of the mobile charge carriers in BaTiO_3_ as electrons rather than oxygen vacancies in previously reported literature. Under thermal activation process, the effective mobile charge carriers can be considered as electrons, holes, and polarons localized in the oxygen vacancies or in the Ti element near the oxygen vacancies. Therefore, the conductivity (*σ*_BT_) of BaTiO_3_ can be expressed as the sum of the conductivities of the three carriers as follows:

5 $${\sigma }_{BT} = {\sigma }_{e}+ {\sigma }_{h}+ {\sigma }_{p},$$

where *σ*_e_, *σ*_h_ and *σ*_p_ are conductivities for electron, hole and polaron, respectively. Here, *σ*_h_ can be negligible because the hole mobility is very low compared to the electron mobility due to the nearly flat valence band^23^. *σ*_BT_ in Eq. (5) can be described as a single Arrhenius equation and employing the effective activation energy as follows:

6 $${\sigma }_{BT} \approx e{\mu }_{e}{N}_{CB}{exp}\left(-\frac{{E}_{C}-{E}_{F}}{{k}_{B}T}\right)+e{N}_{p}{\mu }_{p0}exp\left(-\frac{{E}_{p}}{{k}_{B}T}\right) \approx \left[e{\mu }^{*}{N}^{*}\right]exp\left(-\frac{{E}^{*}\left(T\right)}{{k}_{B}T}\right),$$

where *e* is the electron elementary charge, *μ*_e_ is the electron mobility, *μ*_p0_ is the semi-empirical parameter, *N*_CB_ is the density of states in the conduction band, *N*_p_ is the polaron concentration, *E*_c_, *E*_F_ and *E*_p_ are the conduction band energy, Fermi energy and polaron binding energy, respectively. N*, μ* and E^*^ are the effective parameters of concentration, mobility and activation energy, respectively^23^. The effective activation energy E*(*T*) can be taken on values between 0.3 eV corresponding to *E*_p_^24,25^ and 1.6 eV corresponding to *E*_C_ − *E*_F_ of BaTiO_3_, which is approximately 1/2 *E*_g_. It can be considered that the activation energy value will be a smaller value than 1.6 eV, if the electron polarons contribute more to conduction through a hopping mechanism. However, if oxygen vacancy formation is suppressed by using a donor-rich additive composition, the activation energy values that converge to 1.6 eV can be obtained, as shown in Fig. 5a. Thus, we can consider the band conduction in electronic process as the main conduction mechanism for failure, rather than the electron polaron hopping mechanism by oxygen vacancies. Based on the above the conduction mechanism for failure of typical MLCC, there are two main causes for the mechanism. First, vacancies produce shallow in-gap states below conduction band, e.g. oxygen vacancy related polaron hopping process, donor level, grain boundary, defect by processing error. The energy level for the vacancy is much smaller than *E*_g_/2. The second is the excited electron based on band gap in electronic process. The values of activation energy under thermal activation process for state-of-the-art MLCC products are close to ~ 1.6 eV (~ *E*_g_/2), much higher than these reported in the previous literature, because of recent sophisticated fabrication technologies and a significantly increased donor concentration to enhance lifetime reliability. Thus, the activation energy of MTTF under HALT can be caused by intrinsic behavior of dielectric material in MLCC and harsh evaluation conditions. In addition, the activation energy on the electrical conductivity can be represented as a slope in Arrhenius plot as shown in Fig. 5b. The main factors for conduction are the band gap of the material for intrinsic behavior at high temperature and the donor level for extrinsic behavior at low temperature. In addition, the conduction mechanism for degradation and failure of typical MLCC structure under DC bias is shown in Fig. S1 in Supplementary information.

**Figure 6:**
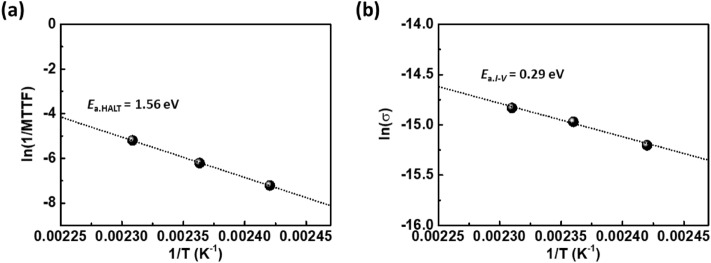
A comparison of the activation energy obtained by (**a**) MTTF under HALT and (**b**) leakage current based conductivity by *I*–*V* curve at a constant electric field of 17.1 V/μm as a function of temperature range from 140 to 160 °C.

For a simple comparison, Fig. 6 shows the activation energy obtained by MTTF under HALT (destructive test) and conductivity by leakage current–voltage (*I*–*V*) curve (nondestructive test) under the same evaluation conditions for electric field and temperature. It is clearly seen that the activation energy on leakage current based conductivity is smaller than that on MTTF under HALT. It is attributed that the activation energy for leakage current based conductivity originates from polaron hopping process and excited electron from donor level, grain boundary, and defect. (extrinsic) Therefore, MTTF under HALT can exhibit more intrinsic behavior due to recent sophisticated fabrication technologies and a significantly increased donor concentration.

**Figure 7:**
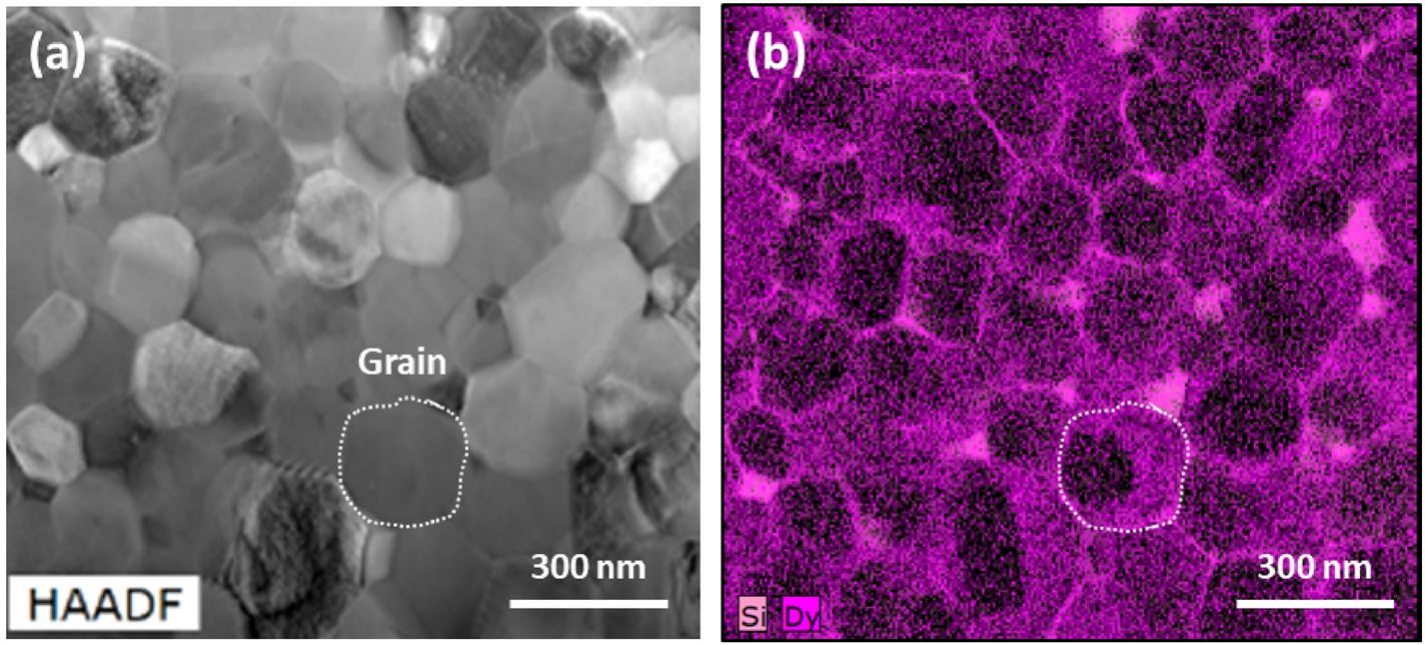
(**a**) High-angle annular dark field (HAADF) and (**b**) energy dispersive spectroscopy (EDS) Dy mapping images of 4.7 μF MLCC.

Furthermore, in case of various additive elements with different valences added to BaTiO_3_, the activation energy for thermal activation process has long been considered in relation to the oxygen vacancies due to similar value to the activation energy (~ 1.0 eV) required for the diffusion of oxygen ions in BaTiO_3_^27–32^. However, a value of 1.0 eV does not necessarily represent the activation energy for the oxygen vacancy, because higher oxygen vacancy migration energy than 1.0 eV for rare earth doped BaTiO_3_, as reported by Cheng et al.^33^, is possible. In terms of resistance degradation over time, the electronic process can also be fully described using the Lloyd model^34,35^ and injection/transport of carriers^24^, as approached by oxygen vacancies^7,29,30^. In addition, the electric field during HALT for an early model of MLCC in literature proposed by Waser et al.^7^ was 0.8 V/μm. It is relatively smaller than the electric field during HALT for the prototype MLCC products. Applying this small electric field to the prototype MLCC, it will almost certainly not fail. The MLCCs used in this study are state-of-the-art products, where the recent sophisticated fabrication technologies are accumulated for high rated voltage and high capacitance, which are composed of a significantly increased donor concentration to enhance lifetime reliability. As shown in Fig. 7, transmission electron microscopy (TEM) images show successful introduction of Dy (rare-earth metal) into grains of dielectric layer in 4.7 μF MLCC as a representative. Since the donor inhibits the formation of oxygen vacancies in BaTiO_3_, the concentration of electron polarons localized in the Ti element is also reduced. Thus, the band conduction behavior of the electrons needs to be considered for conduction mechanism for failure, because the electron polarons can be also excited up to the conduction band as applying high electric field or high temperature. Under the actual operating conditions of the application, it is reasonable to focus the analysis on the electronic processes, and oxygen vacancy migration can be neglected due to the mild conditions below HALT. Therefore, if the electronic process is dominant, a higher bandgap energy is required to enhance the lifetime reliability of MLCC. From the above evidences, we propose that the concepts of electronic processes and oxygen vacancies should be considered together to understand conduction mechanism for failure of BaTiO_3_-based MLCCs in thermal activation processes. It is preferable to utilize activation energy as an indicator for comparing physical properties between compositions and for designing robust MLCC product.

## Conclusion

In summary, we estimated lifetime for three prototype MLCCs (4.7 μF, 0.22 μF, and 10 μF) to obtain the activation energy under thermal activation process. The MTTF of MLCCs was calculated by using the Arrhenius based P–V equation as a method. It was observed that the activation energy shows to be constant in the average value of 1.5 eV for three prototype MLCCs, higher than the activation energy values of ~ 1.0 eV related to the motion or diffusion of oxygen vacancies reported in the previous literature. The activation energy value of ~ 1.5 eV for three prototype MLCCs is close to a half the energy band gap (*E*_g_/2 ≈ 1.6 eV) of BaTiO_3_ obtained from specific environment, where oxygen vacancies are stabilized by external containment such as the effect of rare earth oxide additives. Due to an obvious difference in activation energy values, it difficult to explain the conduction mechanism for failure by only oxygen vacancy migration. Herein, electron behavior based on the recently proposed semiconductor theory is employed as a new attempt to understand failure mechanism of MLCCs under thermal activation process. Additionally, under the actual operating conditions of the application, it is reasonable to focus the analysis on the electronic processes, and oxygen vacancy migration can be neglected due to the mild conditions below HALT. However, oxygen vacancy migration cannot be completely ignored, because it can occur during HALT at the significantly high temperature or electric field. Therefore, both electronic process and oxygen vacancy can be considered as major causes for failure of BaTiO_3_ based MLCC under thermal activation process. It is also preferable to utilize thermal activation energy as an indicator for comparing physical properties between compositions and for future MLCC development with high lifetime reliability.

### Supplementary Information

Supplementary Information.

## Data Availability

The datasets used and/or analysed during the current study available from the corresponding author on reasonable request.

## References

[1] Hong KT (2019). Perspectives and challenges in multilayer ceramic capacitors for next generation electronics. J. Mater. Chem. C.

[2] Xu X (2011). Robust BME class-I MLCCs for harsh-environment applications. IEEE Trans. Ind. Electron..

[3] Park K (2009). The doing effects of intermediate rare-earth ions (Dy, Y, and Ho) on BaTiO_3_ ceramics. J. Korean Ceram. Soc..

[4] Kishi H, Mizuno Y, Chazono H (2003). Base-metal electrode-multilayer ceramic capacitors: Past, Present, and Future perspectives. Jpn. J. Appl. Phys..

[5] Sakabe Y (2002). Effects of rare-earth oxides on the reliability of X7R dielectrics. Jpn. J. Appl. Phys..

[6] Gong H (2016). Synergistic effect of rare-earth elements on the dielectric properties and reliability of BaTiO_3_-based ceramics for multilayer ceramic capacitors. Mater. Res. Bull..

[7] Waser R, Baiatu T, Hardtl KH (1990). DC electrical degradation of perovskite-type titanates: I, ceramics. J. Am. Ceram. Soc..

[8] Tyunina M, Savinov M (2020). Charge transport in epitaxial barium titanate films. Phys. Rev. B.

[9] Tyunina M (2021). Conductivity in ferroelectric Barium Titanate: Electron versus oxygen vacancies. IEEE Trans. Ultra Ferroelectr. Freq. Control.

[10] Kurtz SK, Levinson S, Shi D (1989). Infant mortality, freaks, and wear-out: Application of modern semiconductor reliability methods to ceramic multilayer capacitors. J. Am. Ceram. Soc..

[11] Minford WJ (1982). Accelerated life testing and reliability of high K multilayer ceramic capacitors. IEEE Trans. Compon. Hybrids Manuf. Technol..

[12] Paulsen JL, Reed EK (2002). Highly accelerated lifetesting of base-metal-electrode ceramic chip capacitors. Microelectron. Reliab..

[13] Hernández-López AM (2018). Reliability of X7R multilayer ceramic capacitors during high accelerated life testing (HALT). Materials.

[14] Liu D (2015). Insulation resistance degradation in Ni–BaTiO_3_ multilayer ceramic capacitors. IEEE Trans. Compon. Packag. Manuf. Technol..

[15] Prokopowicz, T. & Vaskas, A. Research and development, intrinsic reliability, subminiature ceramic capacitors. *Final Report*, ECOM-90705-F, NTIS AD-864068 (1969).

[16] Yamamatsu J (1996). Reliability of multilayer ceramic capacitors with nickel electrodes. J. Power Sources.

[17] Randall, M. *et al.* Lifetime modeling of Sub 2 micron dielectric thickness BME MLCC. In *Proceedings 23rd Symposium Passive Components (CARTS USA)* 134–140 (Scottsdale, 2003).

[18] Ashburn, T. & Skamser, D. Highly accelerated testing of capacitors for medical applications. In *SMTA Medical Electronics Symposium* (Anaheim, 2008).

[19] Tateishi T (2019). Effect of La doping on the suppression of insulation resistance degradation in multi-layer ceramic capacitors. Jpn. J. Appl. Phys..

[20] Lee CH, Yoon JR (2022). Effect of La doping on the suppression of insulation resistance degradation in multi-layer ceramic capacitors. J. Ceram. Process. Res..

[21] Yousefian P (2023). Utilizing time domain electrical methods to monitor MLCCs’ degradation. Appl. Phys. Lett..

[22] Elmahgary MG (2023). Optical investigation and computational modelling of BaTiO_3_ for optoelectronic devices applications. Sci. Rep..

[23] Tyunina M (2020). Oxygen vacancies in perovskite oxide piezoelectrics. Materials.

[24] Yamada H, Miller GR (1973). Point defects in reduced strontium titanate. J. Solid State Chem..

[25] Kolodiazhnyi T (2003). Thermoelectric power, hall effect, and mobility of n-type BaTiO_3_. Phys. Rev. B.

[26] Shackelford, J. F. Introduction to materials science for engineers (8th ed.) 460–477 (Pearson, 2015).

[27] Meyer R, Liedtke R, Waser R (2005). Oxygen vacancy migration and time-dependent leakage current behavior of Ba_0.3_Sr_0.7_TiO_3_ thin films. Appl. Phys. Lett..

[28] Maier RA, Randall CA (2016). Low temperature ionic conductivity of an acceptor-doped perovskite: II. Impedance of single-crystal BaTiO_3_. J. Am. Ceram. Soc..

[29] Waser R, Baiatu T, Hardtl KH (1990). DC electrical degradation of perovskite-type titanates: II. Single crystals. J. Am. Ceram. Soc..

[30] Baiatu T, Waser R, Hardtl KH (1990). DC electrical degradation of perovskite-type titanates: III, A model of the mechanism. J. Am. Ceram. Soc..

[31] Chan NH, Sharma RK, Smyth DM (1982). Nonstoichiometry in acceptor-doped BaTiO_3_. J. Am. Ceram. Soc..

[32] Chan NH, Smyth DM (1984). Defect chemistry of donor-doped BaTiO_3_. J. Am. Ceram. Soc..

[33] Cheng X (2023). Defect mechanisms, oxygen vacancy trapping ability in rare-earth doped BaTiO_3_ from first-principles and thermodynamics. J. Am. Ceram. Soc..

[34] Lloyd JR, Liniger E, Shaw TM (2005). Simple model for time-dependent dielectric breakdown in inter- and intralevel low-k dielectrics. J. Appl. Phys..

[35] Wong TKS (2012). Time dependent dielectric breakdown in copper Low-k interconnects: Mechanisms and reliability models. Materials.

