# Supplementary material for: Thermal activation energy on electrical degradation process in BaTiO3 based multilayer ceramic capacitors for lifetime reliability
Source: Sci Rep. 2024 Jan 5;14:616. doi: 10.1038/s41598-024-51254-w (PMC10770127; doi:10.1038/s41598-024-51254-w)
Supplement: Supplementary file 1 — Supplementary Information. [file 41598_2024_51254_MOESM1_ESM.docx]

**Supplementary Information**

**For**

**Thermal Activation Energy on Electrical Degradation Process in BaTiO3 based Multilayer Ceramic Capacitors for Lifetime Reliability**

**Jinsung Chun*^1^, Jungwoo Heo^1^, KyungSoo Lee^2^, Byeong Uk Ye^3^, Byung Sung Kang^1^, Seok-Hyun Yoon*^1^**

*^1^MLCC Development Team, Component Biz. Unit, Samsung Electro-Mechanics Co. Ltd., Suwon, Gyunggi-Do, 16674, Republic of Korea*

*^2^Development QA Team, Quality Assurance Center, Samsung Electro-Mechanics Co. Ltd., Suwon, Gyunggi-Do, 16674, Republic of Korea*

*^3^MLCC Manufacturing Technology Team, Component Biz. Unit, Samsung Electro-Mechanics Co. Ltd., Pusan, KangSeo-Ku, 46754, Republic of Korea*

*(*Corresponding author:* *thousandjs@gmail.com, seokhyun72.yoon@samsung.com)*

**
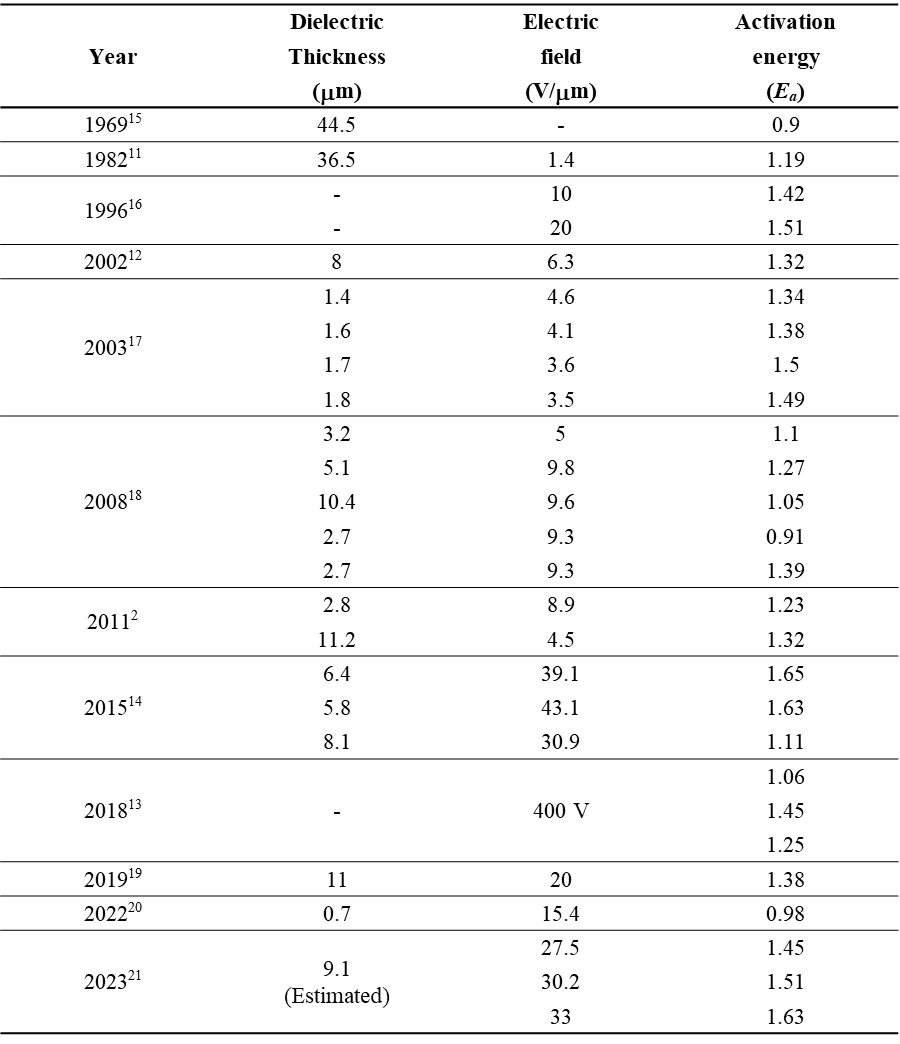
**

**Table S1.** Published year, dielectric thickness, electric field and activation energy from HALT in references^2,11-13,15-21^.


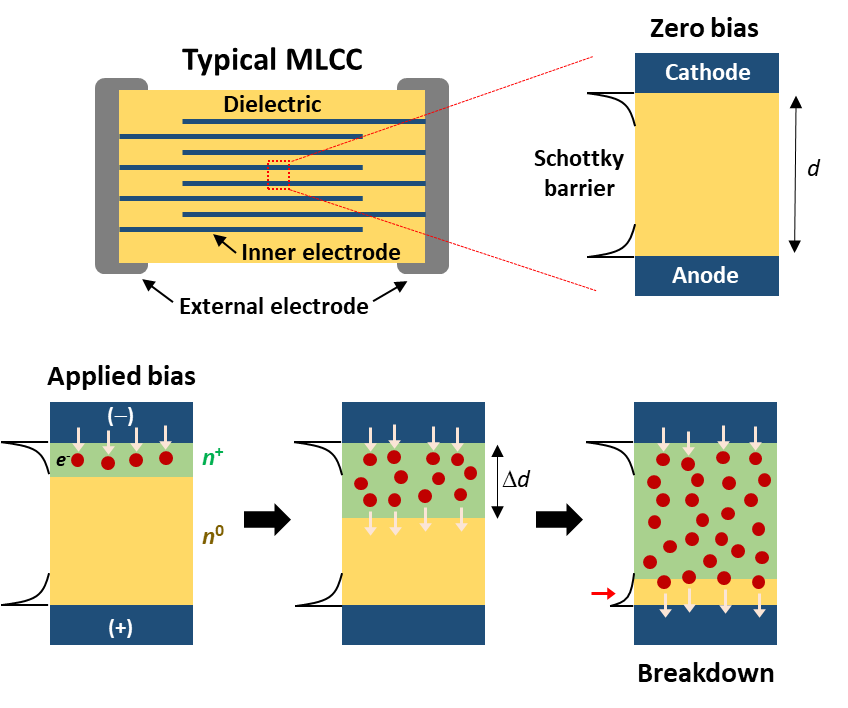


**Figure S1.** Schematic diagram of an electronic process based on the insulation resistance degradation and failure mechanism from cathode to anode of a typical MLCC under DC bias application^2-4^.
